# The human IL-17A/F heterodimer: a two-faced cytokine with unique receptor recognition properties

**DOI:** 10.1038/s41598-017-08360-9

**Published:** 2017-08-21

**Authors:** Arnaud Goepfert, Sylvie Lehmann, Emmanuelle Wirth, Jean-Michel Rondeau

**Affiliations:** Novartis Institutes for BioMedical Research, Novartis Pharma AG, CH-4002 Basel, Switzerland

## Abstract

IL-17A and IL-17F are prominent members of the IL-17 family of cytokines that regulates both innate and adaptive immunity. IL-17A has been implicated in chronic inflammatory and autoimmune diseases, and anti-IL-17A antibodies have shown remarkable clinical efficacy in psoriasis and psoriatic arthritis patients. IL-17A and IL-17F are homodimeric cytokines that can also form the IL-17A/F heterodimer whose precise role in health and disease remains elusive. All three cytokines signal through the assembly of a ternary complex with the IL-17RA and IL-17RC receptors. Here we report the X-ray analysis of the human IL-17A/F heterodimer that reveals a two-faced cytokine closely mimicking IL-17A as well as IL-17F. We also present the crystal structure of its complex with the IL-17RA receptor. Unexpectedly in view of the much higher affinity of this receptor toward IL-17A, we find that IL-17RA is bound to the “F-face” of the heterodimer in the crystal. Using site-directed mutagenesis, we then demonstrate that IL-17RA can also bind to the “A-face” of IL-17A/F with similar affinity. Further, we show that IL-17RC does not discriminate between the two faces of the cytokine heterodimer either, thus enabling the formation of two topologically-distinct heterotrimeric complexes with potentially different signaling properties.

## Introduction

IL-17A and IL-17F are highly pro-inflammatory, “signature cytokines” of effector T helper 17 (Th17) cells, a distinct lineage of CD4^+^ T helper cells of the adaptive immune system that appears to play an important role in clearing pathogens not adequately handled by the Th1 or Th2 immune response^1–4^. IL-17A and IL-17F are also produced by several innate immune cells residing notably in the skin, gut and lung, such as γδ T cells, invariant natural killer T (iNKT) cells, lymphoid-tissue inducer (LTi)-like cells, natural killer (NK) cells, Paneth cells and neutrophils^5, 6^. IL-17A and IL-17F protect the skin and mucosal barriers against infectious agents^6^. Both cytokines act on fibroblasts, epithelial cells and endothelial cells, and play a key role in the recruitment, activation and migration of neutrophils. They induce the secretion of pro-inflammatory cytokines (IL-6, IL-1, TNF), granulopoietic factors (G-CSF, GM-CSF), antimicrobial factors (defensins, S100 proteins)^7^, chemokines (IL-8, CXCL1, CXCL5, CCL2, CCL7) and matrix metalloproteinases (MMP1, MMP3, MMP13). While IL-17A and IL-17F appear to play overlapping, yet distinct roles in host defense against bacterial and fungal infections^8–11^, IL-17A has attracted much attention owing to its implication in several autoimmune diseases, including psoriasis, psoriatic arthritis, ankylosing spondylitis, rheumatoid arthritis, multiple sclerosis and non-infectious uveitis^12–14^.

Human IL-17A and IL-17F map to the same chromosome and are, with 50% amino acid sequence identity, the two most closely related members of the IL-17 protein family^15^. All IL-17 family members (IL-17A, B, C, D, E and F) are homodimeric glycoproteins with a cystine-knot-like fold that lacks one of the canonical disulfides found in bona fide cystine-knot proteins such as TGF-β, bone morphogenetic proteins (BMPs) and nerve growth factors (NGFs), among others. The dimerization mode is distinct from TGF-β and BMPs but like NGFs.

More recently, it was shown that activated human and mouse CD4^+^ T cells also produce a biologically active IL-17A/F heterodimer^16–18^, whose role in health and disease is still largely unknown. Current anti-IL17A therapeutic antibodies such as secukinumab and ixekizumab neutralize the IL-17A homodimer and antagonize the IL-17A/F heterodimer^14, 19^; therefore, it is unclear to what extent, if any, antagonism of the latter contributes to clinical efficacy.

IL-17A, IL-17F and IL-17A/F signaling requires the formation of a heterotrimeric receptor complex comprising IL-17RA and IL-17RC^20–22^. Both IL-17RA and IL-17RC are single pass type I membrane receptors comprising an intracellular signaling motif referred to as the SEFIR domain^23^. This domain is also present in Act1, an adaptor protein with E3 ligase activity that orchestrates initial, membrane-proximal signaling events through homotypic interactions with the SEFIR motif of the IL-17 receptors, leading primarily to TRAF6-dependent activation of the NF-κB and MAP kinase pathways^24, 25^.

Human IL-17RA shows widely different affinities for human IL-17A, IL-17F and IL-17A/F: in comparison to IL-17A, IL-17F binds with at least 100-fold lower affinity to IL-17RA but binds with equally high affinity to IL-17RC^21, 26^. Interestingly, the IL-17A/F heterodimer shows intermediate binding affinity to IL-17RA, and the biological potencies of these three IL-17 cytokines in inducing IL-6 and CXCL1 expression in cell-based assays correlate with their binding affinities toward the IL-17RA receptor^17^.

Structural information is available for the human IL-17A and IL-17F homodimers and their respective binary complexes with the extracellular domain (ECD) of human IL-17RA^27–29^. These structural studies have revealed receptor-induced allosteric changes of the cytokine dimer that explain the formation of asymmetric binary and ternary receptor complexes^29^, in sharp contrast to TGF-β and BMPs which form symmetrical signaling complexes in a 2:2:2 stoichiometry^30–32^.

In comparison to IL-17A and IL-17F, there is a dearth of information on the IL-17A/F heterodimer and its interactions with IL-17RA and IL-17RC, despite its hypothetical therapeutic relevance. Here we present X-ray and site-directed mutagenesis data on human IL-17A/F that provide first detailed insights into the molecular architecture and receptor recognition properties of this heterodimeric IL-17 cytokine.

## Results

### IL-17A/F: a close structural cousin of IL-17A and IL-17F

We produced the full-length human IL-17A/F heterodimer with an APP-tag (EFRHDS) at the N-terminus of the F-chain by co-expression in HEK293 cells, and isolated the heterodimer from the respective homodimers by two successive affinity chromatography steps using anti-tag and anti-IL-17A antibodies. Tetragonal crystals containing one heterodimer in the asymmetric unit and diffracting to 2.3 Å were obtained at pH 7.0, and the structure was solved by molecular replacement (Table 1).

**Table 1:** X-ray data collection and refinement statistics.

Crystal	Free IL-17A/F	IL-17A/F-IL-17RA complex
**Data collection**		
Space group	P4_1_22	P2
*Cell dimensions*		
a, b, c (Å)	87.38, 87.38, 126.32	100.01, 66.11, 104.13
α, β, γ (°)	90.00, 90.00, 90.00	90.00, 89.87, 90.00
Resolution (Å)	2.30 (2.36–2.30)	3.30 (3.39–3.30)
Completeness (%)	99.5 (100.0)	99.1 (99.5)
Redundancy	12.4 (13.2)	3.4 (3.3)
R_merge_	0.130 (1.49)	0.149 (3.48)
I/σ(I)	14.7 (1.8)	5.7 (0.3)
CC^1/2^	99.9 (72.1)	99.7 (18.4)
**Refinement statistics**		
Resolution (Å)	15.00–2.30	66.10–3.30
No. of reflections	22,188	20,143
R_work_	0.205 (0.223)	0.186 (0.257)
R_free_	0.241 (0.254)	0.243 (0.273)
*No. of atoms*		
Protein	1821	7814
Carbohydrate	38	109
Water	96	0
*Mean B-factors (Å* ^2^)		
Protein	52.0	169.1
Carbohydrate	91.0	169.1
Water	49.6	—
*rms deviations*		
Bond length (Å)	0.010	0.010
Bond angle (°)	1.21	1.34
Ramachandran plot	94.4, 5.1, 0.5, 0.0	83.4, 15.2, 1.2, 0.2

Values in parentheses are for the highest resolution shell; values for the Ramachandran plot correspond to most favorable, allowed, generously allowed and disallowed regions.

**Figure 1:**
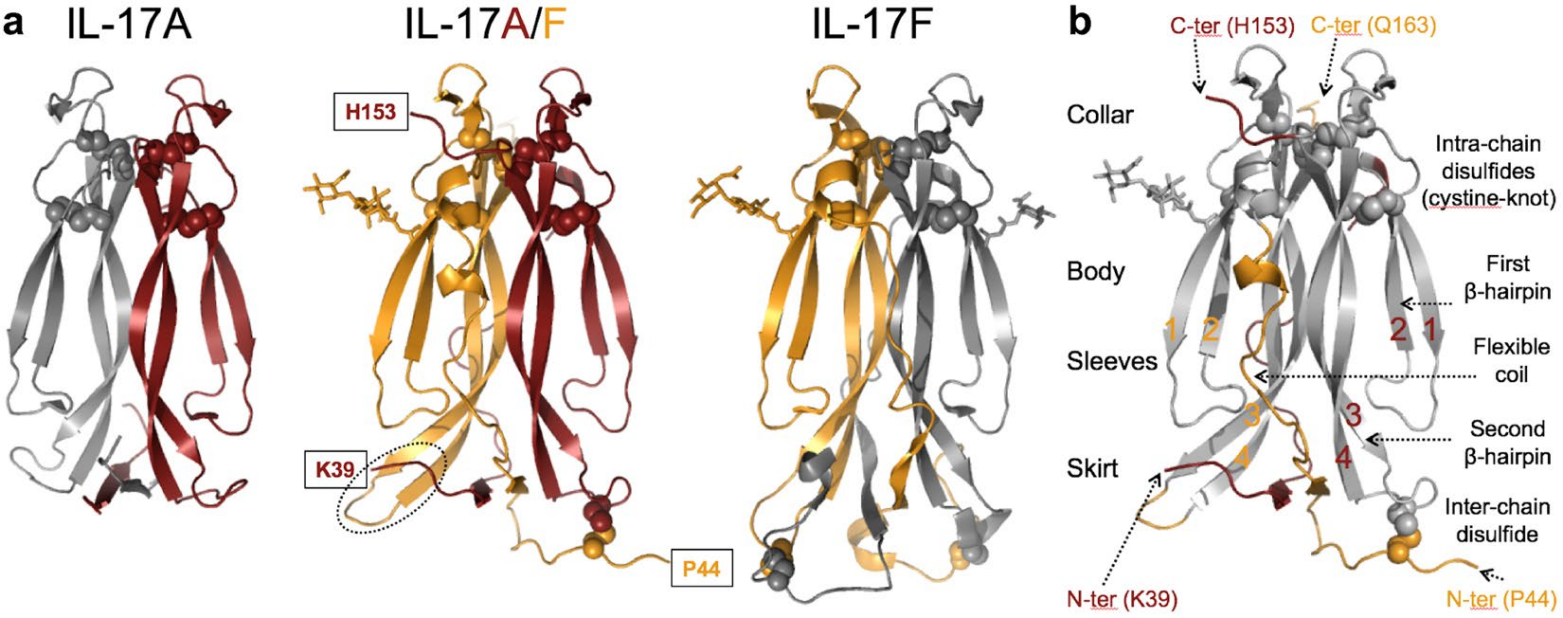
Structure of human IL-17A/F. (**a**) Ribbon diagram of IL-17A/F (center view; A chain, carmine; F chain, orange) and comparison with IL-17A (left, 4hr9.pdb) and IL-17F (right, 1jpy.pdb). Disulfides are represented with spheres. Sugar residues are shown in stick representation. Observed N- and C-termini of IL-17A/F are boxed. The tip of the second β-hairpin of the F-chain is highlighted with a dotted ellipse. (**b**) Upside down orientation emphasizing the analogy between the IL-17 fold and a garment. Structurally-conserved regions in IL-17A/F with respect to the corresponding homodimers are in grey, while non-conserved structural elements are in carmine (A chain) or orange (F chain). The β-strands forming the two β-hairpins of each subunit are numbered sequentially 1 to 4.

The two subunits of the IL-17A/F heterodimer display the expected cystine-knot-like fold made of two β-hairpins connected by intra-chain disulfide linkages. Asn83 of the F-chain carries glycosylation while there is no detectable glycosylation of the A-chain at the predicted site (Asn68). The two polypeptide chains are associated in a head-to-head, parallel fashion, as in the parent homodimeric cytokines (Fig. 1a). Dimerization allows the formation of a small hydrophobic core, which is otherwise lacking in the small, non-globular protein subunits. Both chains retain remarkable structural similarity to their respective homodimeric counterparts, with root-mean square deviations of only 0.9 Å and 0.8 Å, for the A- and F-chain, respectively (81 equivalent Cα atoms in both overlays).

The backbone of the IL-17F homodimer was originally described by Hymowitz *et al*.^27^ as a garment with two sleeves, a collar, a body and a skirt (Fig. 1b). When IL-17A, IL-17F and IL-17A/F are compared, the structurally conserved part comprises the collar (the cystine-knot-like disulfides), the sleeves (first, short β-hairpin) and the body (second, long β-hairpin), while the skirt (N-terminal region forming a small β-sheet with the tip of the second β-hairpin) constitutes a highly variable and substantially disordered region of these three cytokines. The packing of the hydrophobic side-chains within the skirt region is relatively loose, and a hydrophobic cavity is present at the dimer interface, below the N-terminal β-sheet. In addition to the skirt region, the “flexible coil” that connects the N-terminal β-strand to the first β-hairpin is another often-disordered structural element, of poorly conserved sequence and conformation. In IL-17A/F, the tip of the second β-hairpin of the F-chain is roughly orthogonal to the N-terminal β-sheet and therefore does not contribute to it (Fig. 1a).

**Figure 2:**
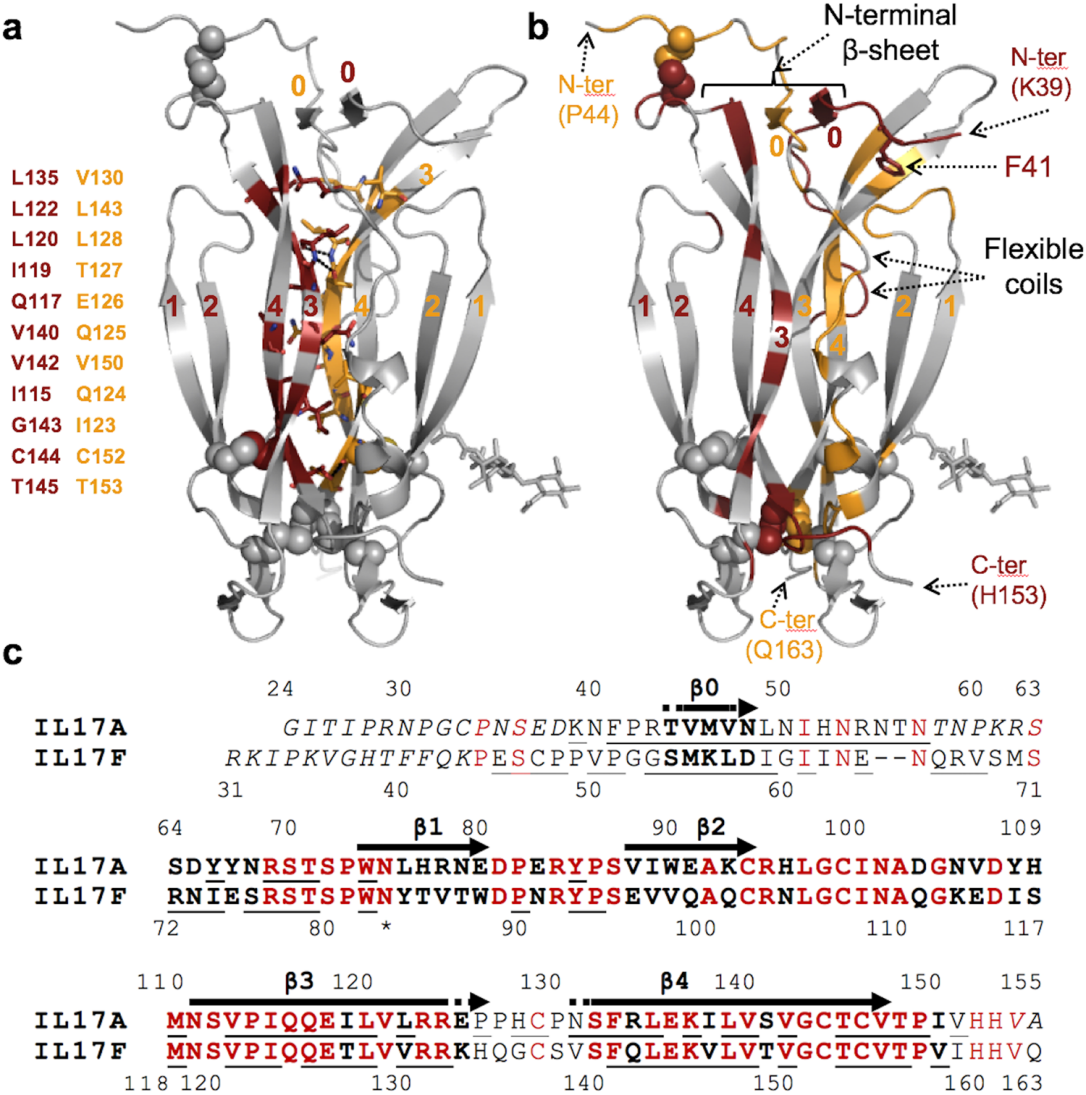
IL-17A/F dimerization interface. (**a**) Core interface of the IL-17A/F heterodimer. The 22 residues forming the core interface, listed on the left, are shown in stick representation, with their positions in the structure shown in carmine and orange for the A- and F-subunit, respectively, while the rest of the heterodimer is in light grey. β-strands are labelled sequentially (0 to 4). (**b**) Non-core interface of the IL-17A/F heterodimer, highlighted using the same color-coding scheme as before. N-termini are labelled. Phe41 (see text) is shown in stick representation. (**c**) Structure-based sequence alignment of the A and F subunits of human IL-17A/F. Disordered residues in the crystal structure are italicized. Structurally equivalent residues are in bold, and conserved amino-acids are highlighted in red. Residues experiencing a reduction (>10 Å^2^) of their solvent accessible surface upon heterodimer formation are underlined. The N-terminal β-strand β0 as well as the first (β1–β2) and second (β3–β4) β-hairpins are indicated with arrows. The observed N-glycosylation site on the F-chain is marked with an asterisk.

The structural variability of the skirt results from the low conservation of N-terminal sequences among IL-17 family members (Fig. 2b), combined with its role in homo- and hetero-dimerization. While up to 1/3 of all residues in IL-17A are disordered^33^, IL-17A/F shows, like IL-17F, more order, with only 13% of residues lacking electron-density (the first 13 and 15 residues of the F- and A-chain, respectively, and residues 58 to 63 of the flexible coil of the A-chain). The inter-chain disulfides are in the skirt. In all currently available IL-17A structures, these were disordered^33^ or engineered out^29, 34, 35^. They are also not defined in the IL-17F complex with IL-17RA^28^, in contrast to the free IL-17F structure^27^. In IL-17A/F, one of the expected two inter-chain disulfides (Cys129A-Cys47F) is well-defined and its assignment agrees with published mass spectrometry data^16^. We note that the segment around Cys47F is involved in crystal contacts which may stabilize the observed conformation.

### IL-17A/F heterodimerization is enabled by a small conserved hydrophobic core and the structural versatility of divergent N-terminal sequences

The dimer interface of IL-17A/F is very large (total buried surface ≈ 5448 Å^2^) and engages approximately 55% of all amino-acid residues. It is comparable in size to those of the IL-17F (≈6824 Å^2^) and IL-17A homodimers (≈4364 Å^2^). The core interface is formed by the second β-hairpin of each subunit (the “body”). These two β-hairpins make contacts to each other that are buried within the protein core and involve 22 residues in total. The majority (64%) of these residues are hydrophobic and highly conserved, with only one non-conservative change, a threonine instead of an isoleucine at position 127 of the F-chain (Fig. 2a,c). Interestingly, the side-chain of Thr127 forms a short, buried H-bond to the A subunit. Nearly 80% of the dimer interface, however, is contributed by poorly conserved sequences forming the skirt region and the flexible coil (Fig. 2b,c). Not too surprisingly, these peptide segments form structural features in IL-17A/F that differ from their counterparts in the respective homodimers (Fig. 1b). Non-conservative changes at the heterodimer interface are thus tolerated through solvent exposure of the side-chains or structural adaptations that exploit new opportunities for inter-chain contacts.

### Structure of IL-17RA bound to the “F-face” of IL-17A/F

The extracellular domain (ECD) of IL-17RA (amino acid residues 33–320) was expressed in GnTI-deficient HEK293S cells. The complex with the IL-17A/F heterodimer was treated with glycosidases and purified by size-exclusion chromatography. The eluted complex had a stoichiometry of one receptor chain per IL-17A/F heterodimer. We found no experimental evidence for the existence of a higher molecular weight complex with two molecules of IL-17RA per IL-17A/F heterodimer. Following crystallization screening, monoclinic crystals diffracting to 3.3 Å and containing two copies of the IL-17RA complex with IL-17A/F in the asymmetric unit were obtained at pH 7.5. The structure was solved by molecular replacement.

**Figure 3:**
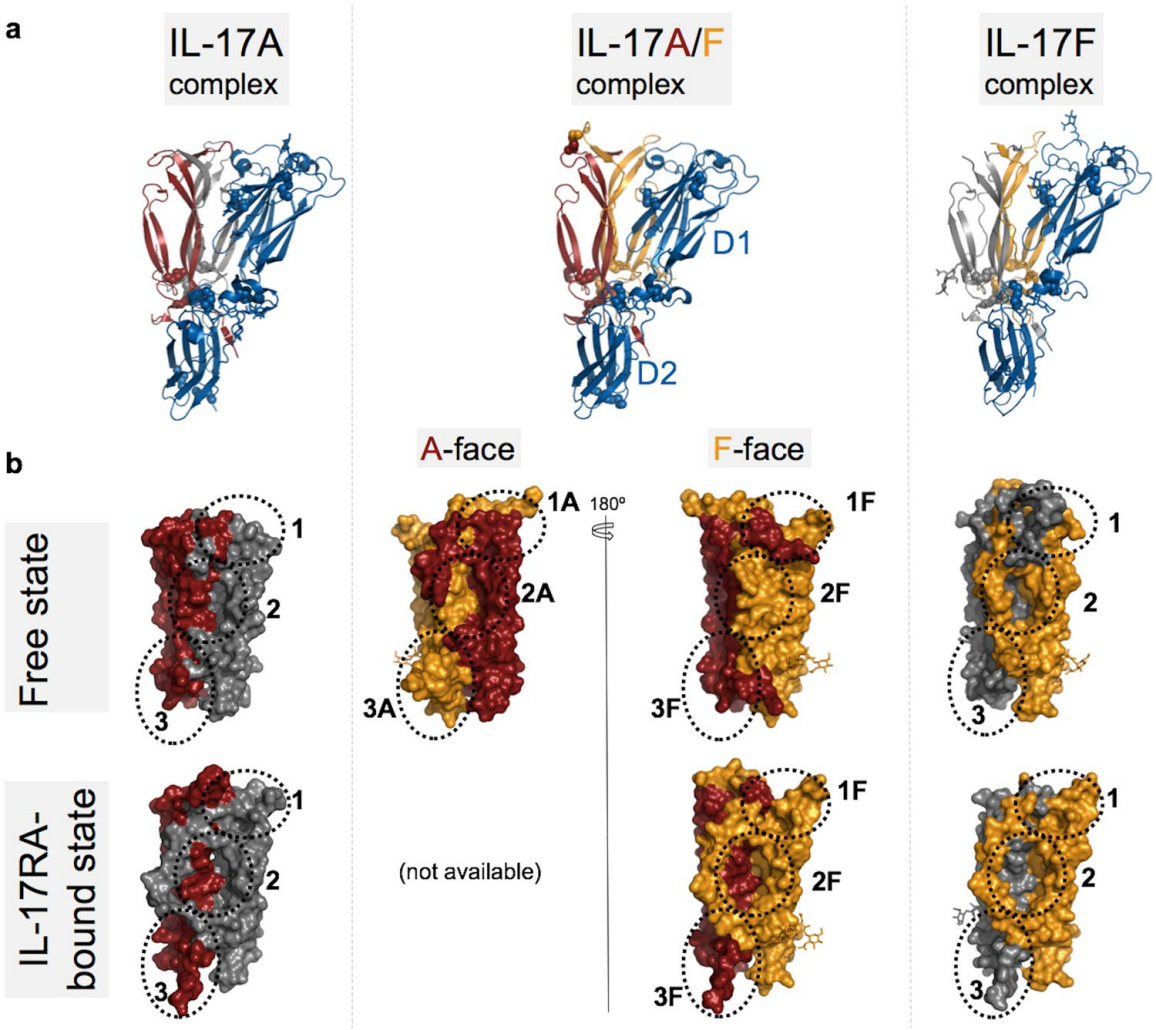
Structural comparison of IL-17RA-cytokine complexes. (**a**) Overall structure of the IL-17A/F complex with IL-17RA (center) compared to the IL-17A (left; 4hsa.pdb) and IL-17F (right; 3jvf.pdb) complexes. The IL-17RA is shown as a blue ribbon, while IL-17A and IL-17F subunits are shown as a carmine or orange ribbon, respectively. The second IL-17 subunit of the homodimeric cytokines is in grey. Disulfide bonds are highlighted as spheres and N-linked glycans are depicted in stick representation. (**b**) Surface representations of IL-17A (left; carmine/grey; 4hr9.pdb), IL-17F (right; orange/grey; 1jpy.pdb) and IL-17A/F (center; carmine/orange) in the free (top row) and IL-17RA-bound states (lower row). For clarity, IL-17RA is omitted from the receptor-bound states. Sites 1–3 are indicated by dotted lines. The two faces of the IL-17A/F heterodimer in the free state are shown (top row, center). The structure of the IL-17A/F complex with IL-17RA bound to the A-face is not known. Note the occlusion of Sites 1–3 on the “F-face” of free IL-17A/F.

The two copies of the IL-17RA complex that make up the asymmetric unit are essentially identical, and overall, highly similar to the published complexes with IL-17A^29^ and IL-17F^28^ (Fig. 3a). Notably, the same stoichiometry of one receptor chain per (hetero)dimer is observed again. The binding interface is similar in size (2373 Å^2^ of total buried surface compared to 2,219 Å^2^ and 2,294 Å^2^ for the IL-17A and IL-17F complexes, respectively) and comprises the same three main interaction sites as in the IL-17A and IL-17F complexes, originally referred to as Site 1–3^28^ (Fig. 3).

Site 1 is in the skirt region and is largely concealed in the structures of the free IL-17A and IL-17F homodimers (Fig. 3b). Significant receptor-induced conformational changes in the skirt region, affecting both subunits, are needed for Site 1 to open. In contrast, Site 2 corresponds to a large, pre-formed cavity which was first described as a “pocket in the garment” by Hymowitz and colleagues^27^.

Site 2 is lined by the two β-hairpins and the flexible coil of one subunit and by the second β-hairpin of the other subunit. In free IL-17F, the flexible coil is ordered and runs along the edge of the pocket, leaving Site 2 largely open (Fig. 3b). In the available structure of free IL-17A, the flexible coil is disordered and therefore Site 2 is also clearly visible. In the free IL-17A/F structure reported here, the flexible coil of the F-subunit is fully ordered and runs across the pocket entrance, hence fully masking Site 2 on this side of the heterodimer. In sharp contrast, the flexible coil of the A chain is partly disordered and runs along the second β-hairpin of the F chain, leaving Site 2 on the other side of the cytokine well accessible to bulk solvent (Fig. 3b).

The structure of the IL-17RA ECD was first described in detail by Garcia and coworkers^28^. It is comprised of two non-canonical fibronectin-III (FnIII) domains connected by a short α-helical linker. The amino-terminal FnIII domain (D1) interacts with Sites 1 and 2 of the cytokine. Both sites are located at the dimer interface, but the relative contributions of the two protein chains are very different. Therefore, the “A-face” can be distinguished from the “F-face” of the IL-17A/F heterodimer, based on which chain contributes most amino-acid residues in these two sites. This situation contrasts with the respective homodimers which offer identical binding sites on either side of the free cytokine.

The carboxy-terminal FnIII domain (D2) of IL-17RA interacts with Site 3. Site 3 is a surface binding site comprising the collar region and C-terminal tail of one cytokine subunit, and part of the flexible coil of the other. Notably, the last five C-terminal residues, which are disordered in the structures of the free cytokines, adopt an extended conformation in the IL-17RA complex and form β-strand/β-strand interactions with the D2 domain of the receptor. The relative contribution of the two cytokine subunits in Site 3 is opposite to that in Sites 1–2.

**Figure 4:**
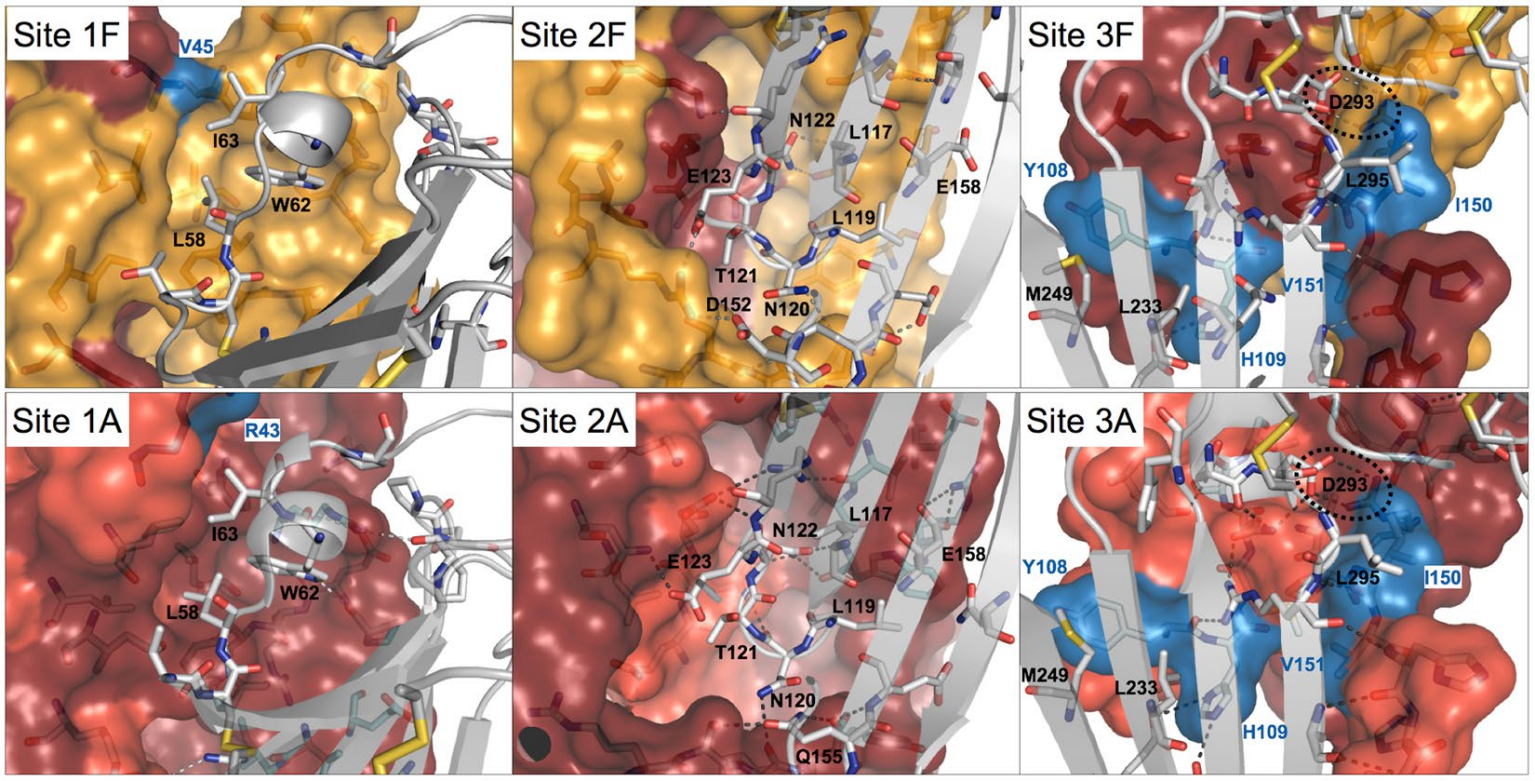
Conserved binding interactions and binding sites in IL-17A, IL17F and IL-17A/F. **Top row:** IL-17A/F complex with IL-17RA. Sites 1F-3F of IL-17A/F are depicted as surface representations, with the A- and F-subunits in carmine and orange, respectively. Non-conserved side-chains with respect to the IL-17F homodimer are in blue. The IL-17RA receptor is shown as a grey ribbon with key side-chains depicted in stick representation. **Bottom row**: IL-17A complex with IL-17RA (4hsa.pdb). Sites 1–3 of IL-17A are shown in surface representation with the 2 subunits in carmine and light red, respectively. Non-conserved side-chains in Sites 1A-3A of IL-17A/F are highlighted in blue. As before, the IL-17RA receptor is shown as a grey ribbon with key side-chains depicted in stick representation. Note the conservation of Site 1A-2A of IL-17A/F with respect to IL-17A.

Based on its much weaker binding affinity toward IL-17F, we were anticipating that the human IL-17RA receptor would bind preferentially to the “A-face” of the IL-17A/F heterodimer. Unexpectedly, the crystal structure revealed binding of the receptor to the “F-face”. This came as a surprising finding also because Site 2A is open while Site 2F is masked by the flexible coil in the free cytokine structure, and a large shift of this structural element is required for receptor binding (Fig. 3). In addition, in the X-ray structure of free IL-17A/F, Site 1F is blocked by several residues of the A-subunit (Lys39 to Arg43) located immediately upstream of the N-terminal β-strand (β0), notably Phe41 whose side-chain occupies the pocket where Trp62 of IL-17RA binds (Fig. 2b, Supplementary Fig. 1). In the receptor complex, these cytokine residues are displaced by the^58^LDDSWI^63^ motif of IL-17RA and become disordered (Fig. 4).

### IL-17A/F: a two-faced IL-17 cytokine closely mimicking both IL-17A and IL-17F

To understand why IL-17RA was bound to the “F-face” of IL-17A/F in the crystal, we first performed an analysis of the conservation of the binding sites.

When we compared the two IL-17RA complexes with IL-17A/F and IL-17F it became immediately apparent that Sites 1F-2F of the heterodimer were highly similar to Sites 1–2 of IL-17F (Fig. 4, Supplementary Table 1). This results naturally from the fact that the F subunit contributes most residues lining Sites 1F-2F of the heterodimer and that all remaining residues contributed by the A-subunit are fully conserved, except for one, Val45 (Met55 in IL-17F), which is located at the rim of Site 1 and is making some hydrophobic contacts to Ile63 of IL-17RA in both complexes. Furthermore, the differences observed in Site 3F were relatively minor, although chain A, rather than F, is the main contributor to this binding site. Notably, the key salt-bridge interactions involving Arg77 and Arg72 of IL-17F and Asp293 of IL-17RA was conserved in the complex with the IL-17A/F heterodimer. The C-terminal tail of the cytokine comprises three amino-acid changes but its binding to IL-17RA mainly involves β-strand/β-strand interactions which are largely sequence-independent. Lastly, two other side-chains that form surface contacts to the receptor are not conserved: Tyr108 (Ile in IL-17F) and His109 (Ser in IL-17F). These mutations have already been investigated by Liu and colleagues^29^ and have been shown to play a minor role, if any, in the affinity difference of IL-17RA toward IL-17A and IL-17F.

The comparison of Sites 1–3 of IL-17A with Sites 1A-3A of IL-17A/F required the generation of a model of IL-17RA bound to the “A-face” of the heterodimer. A crude model was generated by applying to the receptor chain the transformation that would best overlay the F-subunit onto the A-subunit. This model indicated that several structural changes would be required to accommodate the receptor on the “A-face” of the heterodimer: i) a significant conformational change and shift of the loop connecting the β3 and β4 strands ii) a shift/ordering of the flexible coil iii) a shift/ordering of the C-terminal tail. The corresponding structural elements in IL-17A have been found to undergo such structural changes upon IL-17RA binding^29^. When the conservation of residues lining Sites 1A-3A was mapped onto the receptor-bound state of IL-17A, it appeared that Sites 1A-2A of IL-17A/F were essentially identical to their counterparts in IL-17A (Fig. 4, Supplementary Table 1). Again, this is merely a consequence of the fact that the A-subunit is the main contributor to Sites 1A-2A, and all remaining residues provided by the F subunit are fully conserved except for one, Arg43, located at the periphery of Site 1A. The differences in Site 3A were again limited to the two side-chains discussed above (Phe108 to Ile, His109 to Ser), while the key salt-bridge interaction involving Arg69 and the β-strand/β-strand interactions made by the C-terminal tail were maintained.

Hence, IL-17A/F can offer to the IL-17RA receptor two distinct binding surfaces, one of which closely resembles IL-17A, the other IL-17F.

### Neither IL-17RA nor IL-17RC can differentiate between the “A-” and the “F-face” of IL-17A/F

Paradoxically, the analysis above reinforces the view that preferential binding of IL-17RA to the “A-face” of IL-17A/F should follow logically from the mimicry of IL-17A and IL-17F by the two faces of the heterodimer and the well-established binding preferences of this receptor toward the IL-17A and IL-17F homodimers. Notably, we did not identify any structural feature unique to IL-17A/F that could explain disfavored binding of IL-17RA to the “A-face”.

**Figure 5:**
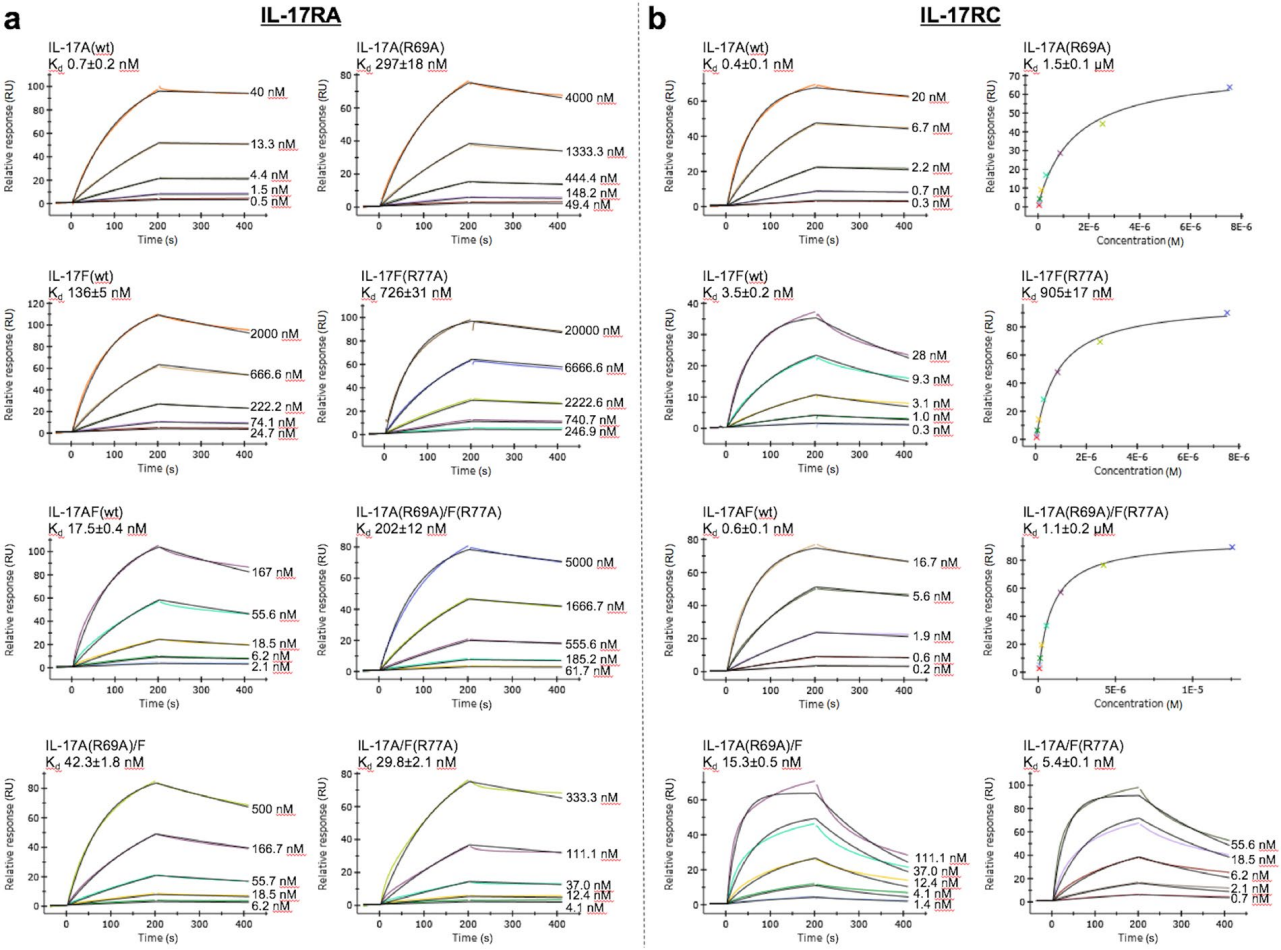
SPR affinity measurements of wild-type and variant IL-17A, IL-17F and IL-17A/F to the immobilized extracellular domain of the IL-17 receptors IL-17RA (**a**) or IL-17RC (**b**). Representative sensorgrams are plotted as response in resonance units (RUs) versus time and shown with colored lines. The concentrations of the injected analytes are indicated on the right of the sensorgrams. The kinetic parameters are calculated using a Langmuir 1:1 binding model with the fitted curves depicted as black lines. For cytokine variants with weaker affinity, the equilibrium dissociation constants were obtained from steady-state analysis by fitting the plot of the response at equilibrium (RUs) as a function of analyte concentration (M). The curve fit is shown as a black line. The indicated K_d_ represents the mean from at least five independent experiments ± the standard error of the mean (sem) and are also summarized in Supplementary Table 2.

To solve this structural riddle, we set out to introduce a point mutation that would strongly reduce receptor binding to the “F-face”, and identified Arg77 of the F-subunit as a potential hot spot at the cytokine-receptor binding interface. To probe the “A-face” of the heterodimer, we also mutated the equivalent residue of the A-chain, Arg69, to alanine. Both side-chains are engaged in a strong salt-bridge interaction with Asp293 of IL-17RA, as revealed by the available crystal structures (Supplementary Fig. 2). The single and double mutants of the heterodimeric cytokine, as well as the corresponding point mutants of the two homodimers, were expressed, purified, and analyzed by surface plasmon resonance (SPR), with the IL-17RA receptor immobilized on the chip surface (Fig. 5a, Supplementary Table 2). The Arg69 to alanine mutation (R69A) caused a nearly 400-fold drop in binding affinity of IL-17A to IL-17RA, while the Arg77 to alanine (R77A) change had a much less pronounced, yet significant effect on the binding of IL-17F (more than 5-fold reduction in affinity). Worthy of note, the salt-bridge interaction to Asp293 of IL-17RA involves only one arginine residue in the case of IL-17A (Arg69) but two arginines in the case of IL-17F (Arg77 and Arg72) (Supplementary Fig. 2), which explains the smaller effect of the point mutation on the F-chain.

If IL-17RA would only recognize the “F-face” of IL-17A/F, the Arg77 to alanine point mutant and the double mutant would be expected to bind with similar affinity, while the Arg69 to alanine mutant would be expected to behave as the wild-type cytokine. Interestingly, while the double mutant of IL-17A/F showed a large drop in binding affinity (11-fold with respect to wt IL-17A/F), the two single mutants showed similar K_D_ values and only a small decrease (approximately 2-fold) in binding affinity in comparison to the wild-type cytokine. Taken together, these site-directed mutagenesis data demonstrate that IL-17RA can bind to the “A-face” as well as to the “F-face” of the IL-17A/F heterodimer, and that, counterintuitively, the binding affinities are very similar. Hence, the crystal structure reported here reveals only one, selected by the crystallization process, of the two, topologically distinct, binary complexes that IL-17RA can form in solution with IL-17A/F.

We then extended our analysis to the IL-17RC receptor. The human IL-17RC extra-cellular domain was expressed, purified and immobilized on the biosensor chip. The three-dimensional structure of IL-17RC is not yet known and the details of its binding interactions to IL-17A, IL-17F and IL-17A/F remain to be elucidated. Therefore, it was not clear whether the Arg69 and Arg77 to alanine mutations would also impact IL-17RC binding. However, these two mutations had a very clear effect on IL-17A and IL-17F binding to IL-17RC, with at least a ≥ 200-fold reduction in binding affinity in both cases (Fig. 5b, Supplementary Table 2). For the IL-17A/F heterodimer, the double mutation showed a dramatic 1800-fold drop in binding affinity compared to the wild-type protein, while the single mutants exhibited intermediate affinities (10- to 25-fold decrease). Notably, the two single mutants again showed similar Kd values (5 nM and 15 nM for the Arg77 and Arg69 mutant, respectively), indicating that IL-17RC, like IL-17RA, can bind either the “A-face” or the “F-face” of the IL-17A/F heterodimer with similar affinities. Nevertheless, and in contrast to the essentially unchanged affinity for IL-17RA, both point mutations significantly reduced affinity toward IL-17RC.

### Structural comparison of the free- and IL-17RA-bound states reveals minimal receptor-induced allosteric changes

**Figure 6:**
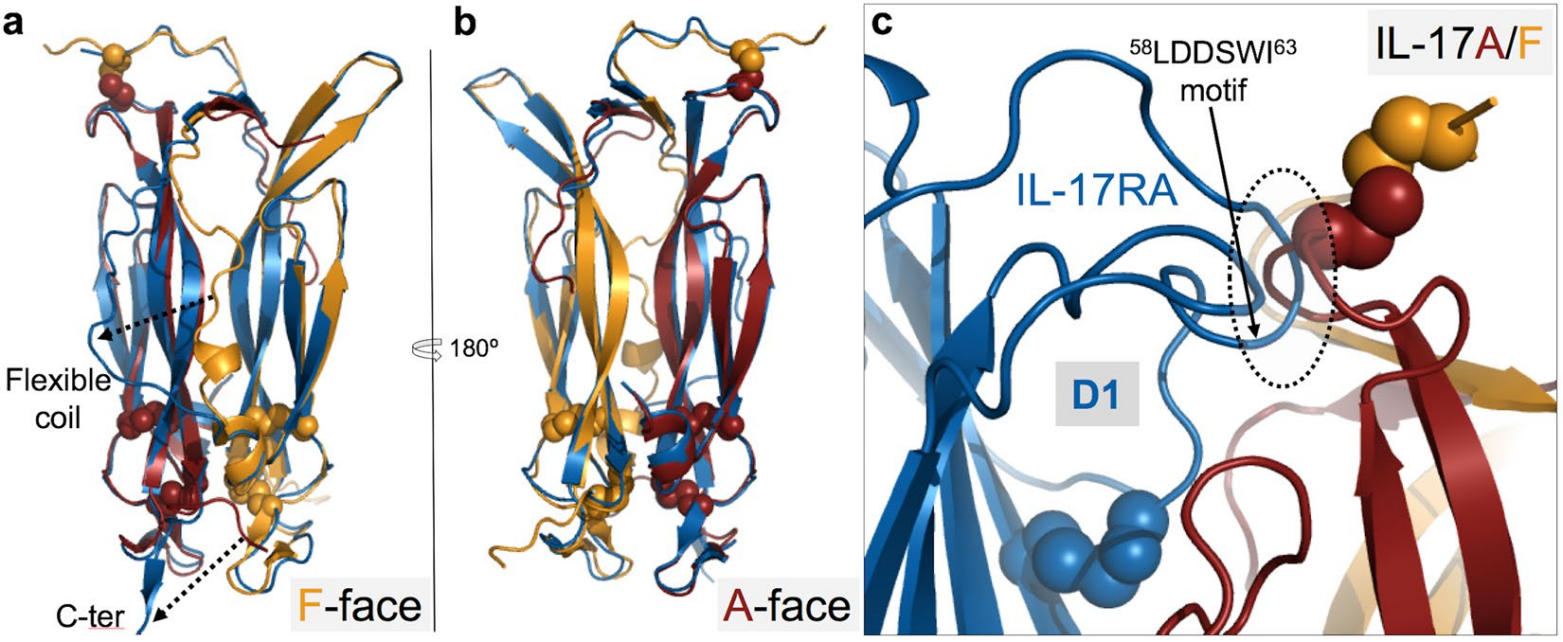
Least square superposition of the free- (carmine/orange ribbon) and IL-17RA-bound states (blue ribbon) of IL-17A/F, using a maximum matching distance of 1.2Å. (**a**) Receptor view of the “F-face” of the cytokine. Note the large conformational changes affecting the flexible coil and C-terminal tail. (**b**) Receptor view of the “A-face”. Note the high similarity of the free- and “F-face”-receptor-bound states. (**c**) Close-up view of a model showing the IL-17RA (blue ribbon) bound to the “A-face” of IL-17A/F. The receptor position was generated by applying the transformation that best superimposes the F-subunit onto the A-subunit. Note the steric hindrance (dotted circle) between the β3–β4 loop of the cytokine and the loop of IL-17RA bearing the key^58^LDDSWI^63^ binding motif.

If IL-17RA can bind to either the “A-face” or “F-face” of IL-17A/F with similar affinity, one may wonder why two molecules of IL-17RA cannot bind simultaneously. This question has been addressed by Liu and colleagues in the context of the IL-17RA complex with IL-17A^29^. We therefore decided to use the same methodology and calculated a structural overlay of the free- and IL-17RA-bound states of IL-17A/F, using a maximum matching distance of 1.2 Å to minimize inaccuracy in the transformation matrix by excluding all residues affected by receptor-induced structural changes. As shown in Fig. 6, while IL-17RA induces significant conformational changes on the “F-face” of IL-17A/F, the “A-face” shows virtually no structural alterations except for small shifts within the skirt region (flexible coil, F-chain N-terminus, and β3-β4 loop of the A-chain). Whether these small changes could play a direct role in preventing the binding of a second copy of the IL-17RA receptor is doubtful. This situation contrasts with the IL-17A complex but is similar to the IL-17F complex (Supplementary Fig. 3, Supplementary Table 3).

However, as already mentioned, receptor binding to the “A-face” would require induced-fit changes, notably to relieve steric hindrance with the β3-β4 loop of the A-chain (Fig. 6c). Other structural elements that would need to undergo conformational changes are the flexible coil and the C-terminal tail.

## Discussion

The impressive clinical success of anti-IL-17 monoclonal antibodies has drawn renewed, considerable attention to IL-17 biology. Our goal in this work was to gain a deeper understanding of the structure and function of IL-17 and more particularly of the human IL-17A/F heterodimer. IL-17A/F is expressed in higher amounts than IL-17A by differentiated Th17 cells, which may compensate, at least in part, for its weaker affinity toward the IL-17RA receptor, thus suggesting a potential biological role in T-cell mediated immune responses^18^. Moreover, IL-17A/F is also antagonized by current anti-IL-17A antibodies^14, 19^ and it may thus have therapeutic relevance for the corresponding clinical indications, although data in support of this view are still lacking, even a decade after the discovery of the heterodimer.

Our X-ray analysis shows how one IL-17A and one IL-17F chain can assemble into a heterodimer while maintaining high structural similarity to the respective homodimeric forms, except for their non-conserved amino-terminal extension which constitutes the structurally variable “skirt”. Hence, the new crystal structure reinforces the view that the IL-17 family is made of a conserved, structurally stable cystine-knot-like “body” extended by a versatile, flexible and rather unstable “skirt”. Further evidence for the structural instability of the skirt emerged from recent drug discovery activities targeting IL-17A^34, 35^.

The structure of the IL-17A/F complex with IL-17RA confirms the binding stoichiometry of only one receptor chain per (hetero)dimer, already observed with IL-17F and IL-17A^28, 29^. This is worthy of note as the exact stoichiometry of IL-17 signaling complexes remains to be fully established^28, 36^. Interestingly, IL-17RA induces conformational changes and forms binding contacts to the “F-face” of IL-17A/F that are very similar to those reported for IL-17F^28^, while the “A-face” of the heterodimer bears close resemblance to IL-17A. Therefore, IL-17A/F can be regarded as a “two-faced” IL-17 cytokine which closely mimics both IL-17A and IL-17F, but has distinct expression levels and biological potency.

Paradoxically, the intermediate affinity of IL-17RA toward either face of the IL-17A/F heterodimer indicates a drop in binding affinity toward the “A-face”, compared to IL-17A, and an increase in binding affinity toward the “F-face”, compared to IL-17F, despite the close structural similarities underlined here above. The structural basis for this behavior is not clear. In sharp contrast to the mouse orthologues, human IL-17RA exhibits much reduced affinity toward IL-17F compared to IL-17A, and attempts have been made to pin down the residues involved. Published data to date indicate that non-conserved residues which are in direct contact to the receptor only partially account for the observed differences in binding affinity^29^. Our finding that both faces of IL-17A/F bind equally well IL-17RA further suggests that other non-conserved residues, located at a distance from the three main binding sites but affected by induced-fit changes, influence the overall free energy of binding. Alternatively, or in addition, the energetic cost of the structural transitions affecting the poorly conserved flexible coil and C-terminal tail upon IL-17RA binding may also be a differentiating factor contributing to the observed affinity differences.

Unlike other homodimeric cytokines which form symmetrical receptor complexes^30–32, 37^, both IL-17A and IL-17F form asymmetrical binary complexes with IL-17RA and, presumably, asymmetrical signaling complexes as well^28, 29, 36^. It has been proposed that symmetrical binding of IL-17RA is precluded by steric hindrance between the D2 domains of the two receptor chains^28^, or, alternatively, by receptor-induced allosteric changes within the “skirt” region of the cytokine that subsequently favor engagement of IL-17RC over that of a second IL-17RA^29^. The latter proposal emerged from X-ray studies of IL-17A, that have revealed dramatic structural differences between the free- and IL-17RA-bound states of the cytokine^29^, which were not seen in previous X-ray studies with IL-17F^28^. Being asymmetrical itself, IL-17A/F cannot form any symmetrical complex with IL-17RA and IL-17RC. Nevertheless, it binds, like its homodimeric counterparts, only one copy of the IL-17RA receptor chain rather than two, and this is not a consequence of preferential binding of the receptor to the “A-” or to the “F-face” of the heterodimer. Rather, as demonstrated by our SPR data with IL-17A/F mutants, IL-17RA can bind to the “A-face” as well as to the “F-face” of IL-17A/F with similar affinity. While the X-ray structure of the IL-17RA complex presented here revealed the receptor bound to the “F-face” of the heterodimer, the other complex with IL-17RA bound to the “A-face” exists as well, as demonstrated by our mutagenesis study. We took advantage of the Arg77 to alanine (F-chain) variant of IL-17A/F to produce more homogeneous samples of this other complex for crystallization and succeeded in obtaining large, nice-looking crystals. Unfortunately, and despite best efforts, these crystals did not diffract well enough to allow structure determination.

Although we applied the methodology proposed by Liu and colleagues^29^ for optimal superposition of the free- and receptor bound states of IL-17A/F, our structural overlay did not reveal any significant receptor-induced allosteric changes on the “A-face” that could readily explain disfavored binding of a second IL-17RA. Rather, our IL-17A/F data are very much in line with the IL-17F case, which did not provide any evidence for allosteric changes of the kind and magnitude seen in the IL-17A case either^28^. A potential shortcoming of such structural comparisons is that the crystal structures of neither the free- nor of the liganded-state may be fully representative of the solution state(s). Also, we note that the structure of IL-17A was obtained from a protein construct lacking the first 10 amino-acids as well as both interchain disulfides, which might allow conformational states not accessible to the wild-type protein.

On the other hand, our data do not strengthen the case for the “steric hindrance hypothesis” either^28^. Our model of the IL-17A/F heterodimer with IL-17RA bound to both sides does not show any steric hindrance between the two receptor chains (Supplementary Fig. 4). Therefore, we believe that an allosteric mechanism must be responsible for the binding of only one IL-17RA to IL-17A/F, but that this mechanism may be more subtle in the IL-17F and IL-17A/F cases than was suggested by the IL-17A work so far. A hint at a possible allosteric mechanism is provided by this model, which shows that a significant change in the conformation of the β3-β4 loop of the A-subunit is required for receptor binding to the “A-face” (Fig. 6c). This loop is directly in contact with the N-terminal region of the F-chain and is connected to it by one of the inter-chain disulfides. Therefore, any receptor-induced conformational switch of the β3-β4 loop on the “A-face” is bound to induce further structural alterations propagating into the N-terminal β-sheet and further into the F-subunit, inevitably affecting the “F-face” of the cytokine. Hence, there is little doubt that a structural cross-talk is at play between the two faces of IL-17A/F, more particularly in the structurally-unstable “skirt” region. Thus, our results qualify, but are otherwise largely in support of, the proposal that IL-17RA binding induces structural asymmetry in the “skirt” region of IL-17 cytokines that prevents simultaneous binding of two copies of this receptor^29^.

**Figure 7:**
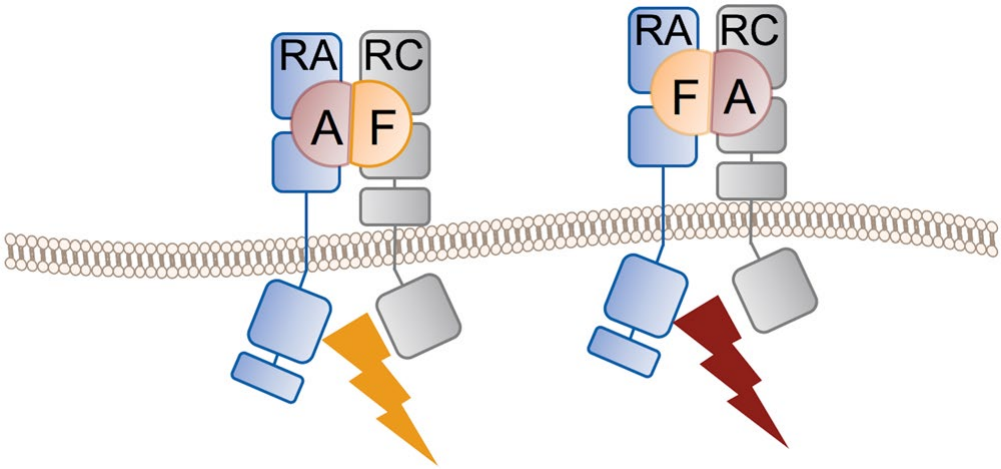
Schematic representation of the two, topologically distinct, signaling complexes potentially formed by the human IL-17A/F heterodimer and the IL-17RA and IL-17RC receptor chains. Whether both complexes are competent for signaling and whether they trigger the same downstream signaling events is not known.

Interestingly, the IL-17RC receptor too does not discriminate between the “A-face” and the “F-face” of the heterodimer. Therefore, IL-17RC may also form two topologically distinct binary complexes with IL-17A/F. Taken together, these data also suggest that the IL-17A/F heterodimer may form two topologically distinct signaling complexes with IL-17RA and IL-17RC, in contrast to IL-17A and IL-17F (Fig. 7). This raises the interesting possibility that these two heterotrimeric complexes may have distinct signaling properties. Therefore, further investigations are warranted to firmly establish whether these two complexes truly exist and trigger similar or distinct downstream signaling events.

In summary, the results reported here provide detailed structural information on the human IL-17A/F heterodimer that should greatly facilitate future studies aimed at improving our understanding of the biology of this peculiar IL-17 family member and its potential role in chronic inflammatory or autoimmune diseases. In particular, they may facilitate the design of IL-17A/F –specific antagonists, or, alternatively, IL-17A- or IL-17F-specific drugs.

## Methods

### Cloning

The full-length IL-17A (amino acid residues 1–155 of Uniprot entry Q16552) using the native signal peptide and IL-17F (amino acid residues 31–163 of Uniprot entry Q96PD4) fused to the Myeloid cell surface antigen CD33 signal peptide (UniProt ID: P20138-1) were cloned into a pCI-derived co-expression vector. To facilitate purification, a synthetic peptide with the amino acid sequence EFRHDS derived from the human Amyloid Precursor protein (APP6-tag) was fused N-terminally to the IL-17F chain. The Arg69 to alanine and Arg77 to alanine mutations on the IL-17A and IL-17F chains, respectively, were generated by site-directed mutagenesis. For crystallization, the full-length extracellular domain (ECD) of IL-17RA (amino acid residues 33-320 of UniProt entry Q96F46) fused to the CD33 protein signal peptide and a C-terminal APP6-Avi-tag was cloned in a pRS5-derived vector. The full-length ECD domain of IL-17RC (amino acid residues 21-467 of UniProt entry Q8NAC3-2) fused to the CD33 protein signal peptide and a C-terminal APP6-tag was cloned in a pRS5-derived vector.

### Protein expression and purification

IL-17A/F constructs were transiently expressed in human embryonic kidney (HEK) 293S cells. The day prior transfection, the cells were diluted with V3 medium (Novartis medium, patent: WO 2011/134920 A1) to a final concentration of 2 × 10^6^ cells.ml^−1^. The next day, cells were centrifuged and diluted with fresh medium to 3 × 10^6^ cells.ml^−1^ and DNA was introduced into host cells by transfection with polyethylenimine (PEI) using 1 µg of DNA per 1 ml of culture and a 2:1 ratio of PEI to DNA (w/w). The cells were incubated on an orbital shaking platform at 37 °C with 5% CO_2_ at a speed of 100 rpm for 3h. Cells were then diluted 2 times to ∼1.5 × 10^6^ cells.ml^−1^ and incubated for 6 days. The same expression protocol was used for the IL-17RA and IL-17RC ECD constructs but using GnTI-deficient HEK293S cells and Freestyle™ 293 medium (Invitrogen).

Cells expressing IL-17A/F were harvested and the supernatant concentrated. Co-expression of IL-17A and IL-17F lead to the production of the IL-17A/F heterodimer but also of the IL-17A and IL-17F homodimers. The three isoforms were separated by affinity chromatography using a column containing NHS-activated resin coupled to an anti-Amyloid Precursor Protein (APP) antibody. While the IL-17A homodimer was in the flow-through, the IL-17A/F heterodimer and the IL-17F homodimer were retained on the column and were eluted with a one-step gradient of 0.1 M glycine (pH 2.7). To immediately restore physiological pH, 1.0 M of Tris (pH 8.5) was added to the eluted fractions. IL-17A was further purified by affinity chromatography using the human anti-IL-17A monoclonal antibody XAB4 (WO 2014/122613 A1) and eluted as described for the anti-APP antibody column. IL-17A/F was separated from IL-17F homodimers by affinity chromatography using the same anti-IL-17A monoclonal antibody. The isolated IL-17A, IL-17A/F and IL-17F proteins were then concentrated using vivaspin and further purified by size-exclusion chromatography with a Superdex-75 10/300 equilibrated with either 10 mM Tris (pH 7.5), 50 mM NaCl or PBS (pH 7.4), 1 mM EDTA, for crystallization or SPR measurements, respectively.

For the IL-17RA ECD construct, the same affinity purification using an anti-APP antibody was used, followed by size exclusion chromatography with a Superdex-200 26/600 equilibrated with either 10 mM Tris (pH 7.5), 100 mM NaCl or PBS (pH 7.4) for crystallization or SPR measurements, respectively. For SPR measurements, specific biotinylation of the Avi-tag was performed using E. coli biotin ligase (BirA) while IL-17RA was trapped on the anti-APP antibody column.

The same purification protocol was used for the IL-17RC ECD except for an additional intermediate purification step. After affinity purification, the protein was applied to a Resource-Q anion exchange column (Amersham Biosciences) and eluted with a linear gradient of NaCl. Peak fractions were concentrated and further purified by gel filtration chromatography using a Superdex-200 16/600 and PBS (pH 7.4).

The IL-17A/F-IL-17RA complex was formed by mixing the two proteins with a 1.2-fold molar excess of cytokine followed by treatment with PNGaseF (New England BioLabs, MA, USA) and Endoglycosidase H (Sigma-Aldrich, MO, USA) overnight at 25 °C and purification by size-exclusion chromatography with a Superdex-200 10/300 equilibrated with 10 mM Tris (pH 8.0) and 50 mM NaCl.

### Crystallization

The purified protein samples were concentrated to 13.9 and 7 mg ml^−1^ for the IL-17A/F heterodimer and the corresponding complex, respectively.

All crystals were obtained at 20 °C using the sitting-drop vapor diffusion method after mixing 0.2 μl protein solution with 0.2 μl reservoir solution. IL-17A/F crystallized from 0.1 M Bis-Tris-propane (pH 7.0), 0.5 M succinic acid (pH 7.0), and the cytokine-receptor complex from 0.1 M HEPES (pH 7.5), 15% (w/v) PEG MME 5000, 0.05 M ammonium acetate. Crystals of the complex were cryo-protected in crystallization solution supplemented with 10% glycerol, whereas no cryo-protection was needed for IL17A/F crystals. All crystals were flash-frozen into liquid nitrogen.

### Structure determination and refinement

All diffraction data were collected at 100 K at beamline X10SA (PXII) of the Swiss Light Source (λ = 1.0 Å) with a PILATUS pixel detector, and processed using XDS^38^. The crystals of the IL-17A/F complex with IL-17RA diffracted only to medium resolution. With a fixed <I/σ(I)> cutoff of 1.0 or CC1/2 cutoff of 50% we would have cut our data at 3.62 Å or 3.57 Å resolution, respectively. Instead, all data to 3.30 Å were included, based on statistical significance^39^. The structures were determined by molecular replacement with PHASER^40^. For the IL-17A/F heterodimer, a high-resolution structure of IL-17A (solved *in house*) was used as search model, while the structure of the IL-17RA complex with IL-17A (PDB code 4hsa.pdb^29^) was used as search model for the structure solution of the IL-17RA complex with IL-17A/F. For all structure determinations, several rounds of iterative model building and refinement were performed using Coot^41^ and AutoBuster^42^, respectively. 5% of the diffraction data were excluded from refinement and used for cross-validation. The geometry of the final model was assessed using Procheck^43^. For the IL-17A/F complex with IL-17RA, paired refinement tests were run which confirmed that a higher quality model was obtained by cutting the data at 3.30 Å rather than 3.57 Å or 3.62 Å (Supplementary Table 4). All figures were generated with Pymol^44^. Protein interfaces were analyzed with PISA^45^. Structural overlays were calculated with Lsqman^46^.

### SPR

Binding affinities between IL-17 mutants and the IL-17RA and IL-17RC receptors were determined by surface plasmon resonance meaurements using a Biacore 8K biosensor (GE Healthcare). Protein concentration were quantified by monitoring UV absorbance at 210nm using High Performance Liquid Chromatography (HPLC). Biotinylated IL-17RA ECD was diluted in PBS (pH 7.4), with 3 mM EDTA and 0.05% Tween20 and immobilized to a level of approximately 500RU on a streptavidin coated sensor chip (SA chip, GE Healthcare) that has been conditioned with 1 minute injections of 1 M NaCl in 50 mM NaOH. IL-17RC ECD was immobilized onto flow cells of a CM4 sensor chip (GE Healthcare) using standard amine coupling procedure to a level of approximately 500 RU. For the measurements, all wt and mutant cytokines were diluted in PBS (pH 7.4), with 3 mM EDTA and 0.05% Tween20, to a starting concentration of about 50 times the Kd and serial dilutions in three-fold increments were made. To determine kinetic constants, sensorgrams were collected at 20 °C with a flow rate of 30 µl/min. Each protein was loaded for 200 s with a dissociation time of 200 s. The sensor chip surface was regenerated between runs with 3.0 M MgCl_2_. All experiments were performed at least five times independently. All data were analyzed with the Biacore 8 K evaluation software and fitted to a 1:1 Langmuir binding model.

### Accession codes

Protein Data Bank: coordinates and structure factors, 5N92: IL-17A/F, 5NAN:IL-17RA complex with IL-17A/F.

## Electronic supplementary material

Supplementary information

## References

[1] Harrington LE (2005). Interleukin 17-producing CD4^+^ effector T cells develop via a lineage distinct from the T helper type 1 and 2 lineages. Nat. Immunol..

[2] Park H (2005). A distinct lineage of CD4 T cells regulates tissue inflammation by producing interleukin 17. Nat. Immunol..

[3] Korn T, Bettelli E, Oukka M, Kuchroo VK (2009). IL-17 and Th17 cells. Annu. Rev. Immunol..

[4] Kara EE (2014). Tailored immune responses: novel effector helper T cell subsets in protective immunity. PLOS Pathog..

[5] Reynolds JM, Angkasekwinai P, Dong C (2010). IL-17 family member cytokines: regulation and function in innate immunity. Cytokine Growth Factor Rev..

[6] Cua DJ, Tato CM (2010). Innate IL-17-producing cells: the sentinels of the immune system. Nat. Rev. Immunol..

[7] Kolls JK, McCray PB, Chan YR (2008). Cytokine-mediated regulation of antimicrobial proteins. Nature Rev. Immunol..

[8] Yang XO (2008). Regulation of inflammatory responses by IL-17F. J. Exp. Med..

[9] Ishigame H (2009). Differential roles of interleukin-17A and -17F in host defense against mucoepithelial bacterial infection and allergic responses. Immunity.

[10] Puel A (2011). Chronic mucocutaneous candidiasis in humans with inborn errors of interleukin-17 immunity. Science.

[11] Iwakura Y, Ishigame H, Saijo S, Nakae S (2011). Functional specialization of interleukin-17 family members. Immunity.

[12] Miossec P, Korn T, Kuchroo VK (2009). Interleukin-17 and type 17 helper T cells. N. Engl. J. Med..

[13] Miossec P, Kolls JK (2012). Targeting IL-17 and T_H_17 cells in chronic inflammation. Nature Rev. Drug Discov..

[14] Patel DD, Kuchroo VK (2015). Th17 cell pathway in human immunity: lessons from genetics and therapeutic interventions. Immunity.

[15] Moseley TA, Haudenschild DR, Rose L, Reddi AH (2003). Interleukin-17 family and IL-17 receptors. Cytokine Growth Factor Rev..

[16] Wright JF (2007). Identification of an interleukin 17F/17A heterodimer in activated human CD4^+^ T cells. J. Biol. Chem..

[17] Chang SH, Dong C (2007). A novel heterodimeric cytokine consisting of IL-17 and IL-17F regulates inflammatory responses. Cell Res..

[18] Liang SC (2007). An IL-17F/A heterodimer protein is produced by mouse Th17 cells and induces airway neutrophil recruitment. J. Immunol..

[19] Liu L (2016). Generation and characterization of ixekizumab, a humanized monoclonal antibody that neutralizes interleukin-17A. J. Inflamm. Res..

[20] Toy D (2006). Cutting edge: interleukin 17 signals through a heteromeric receptor complex. J. Immunol..

[21] Wright JF (2008). The human IL-17F/IL-17A heterodimeric cytokine signals through the IL-17RA/IL-17RC receptor complex. J. Immunol..

[22] Hu Y (2010). IL-17RC is required for IL-17A- and IL-17F-dependent signaling and the pathogenesis of experimental autoimmune encephalomyelitis. J. Immunol..

[23] Novatchkova M (2003). The STIR-domain superfamily in signal transduction, development and immunity. Trends Biochem. Sci..

[24] Chang SH, Park H, Dong C (2006). Act1 adaptor protein is an immediate and essential signaling component of interleukin-17 receptor. J. Biol.Chem..

[25] Qian Y (2007). The adaptor Act1 is required for interleukin 17-dependent signaling associated with autoimmune and inflammatory disease. Nat. Immunol..

[26] Kuestner RE (2007). Identification of the IL-17 receptor related molecule IL-17RC as the receptor for IL-17F. J. Immunol..

[27] Hymowitz SG (2001). IL-17s adopt a cysteine knot fold: structure and activity of a novel cytokine, IL-17F, and implications for receptor binding. EMBO J..

[28] Ely KE, Fischer S, Garcia KC (2009). Structural basis of receptor sharing by interleukin 17 cytokines. Nat. Immunol..

[29] Liu S (2013). Crystal structures of interleukin 17A and its complex with IL-17 receptor A. Nat. Commun..

[30] Allendorph GP, Choe S (2006). Structure of the ternary signaling complex of a TGF-beta superfamily member. Proc. Natl. Acad. Sci. USA.

[31] Groppe J (2008). Cooperative assembly of TGF-beta superfamily signaling complexes is mediated by two disparate mechanisms and distinct modes of receptor binding. Mol. Cell..

[32] Townson SA (2012). Specificity and Structure of a High Affinity Activin Receptor-like Kinase 1 (ALK1) Signaling Complex. J. Biol. Chem..

[33] Gerhardt S (2009). Structure of IL-17A in complex with a potent, fully human neutralizing antibody. J. Mol. Biol..

[34] Liu S (2016). Inhibiting complex IL-17A and IL-17RA interactions with a linear peptide. Sci. Rep..

[35] Liu S (2016). Binding site elucidation and structure guided design of macrocyclic IL-17A antagonists. Sci Rep..

[36] Gaffen SL (2009). Structure and signaling in the IL-17 receptor family. Nat. Rev. Immunol..

[37] Gong Y (2008). Crystal structure of the neurotrophin-3 and p75NTR symmetrical complex. Nature.

[38] W. Kabsch, Automatic processing of rotation diffraction data from crystals of initially unknown symmetry and cell constants. *J. Appl. Crystallogr*. **26**, 795–800 (1993).

[39] P. A. Karplus, K. Diederichs, Linking Crystallographic model and data quality. *Science* **336**, 1030–1033 (2012). 10.1126/science.1218231 PMC3457925 22628654

[40] A. J. McCoy, R. W. Grosse-Kunstleve, P. D. Adams, M. D. Winn, L. C. Storoni, R. J. Read, Phaser crystallographic software. *J. Appl. Crystallogr*. **40**, 658–674 (2007). 10.1107/S0021889807021206 PMC2483472 19461840

[41] P. Emsley, B. Lohkamp, W. G. Scott, K. Cowtan, Features and development of Coot. Acta Crystallogr. D, *Biol. Crystallogr*. **66**, 486–501 (2010). 10.1107/S0907444910007493 PMC2852313 20383002

[42] G. Bricogne, E. Blanc, M. Brandl, C. Flensburg, P. Keller, W. Paciorek, P. Roversi, A. Sharff, O. S. Smart, C. Vonrhein, T. O. Womack, BUSTER version 2.11.2. Cambridge, United Kingdom: Global Phasing Ltd (2011).

[43] R. A. Laskowski, M. W. MacArthur, D. S. Moss, J. M. Thornton, PROCHECK: a program to check the stereochemical quality of protein structures. *J. Appl. Crystallogr*. **26**, 283–291 (1992).

[44] W. L. DeLano, Pymol Molecular Graphics System (DeLano Scientific, San Carlos, California, 2002).

[45] E. Krissinel, K. Henrick, Inference of macromolecular assemblies from crystalline state. *J. Mol. Biol*. **372**, 774–797 (2007). 10.1016/j.jmb.2007.05.022 17681537

[46] G. J. Kleywegt, Use of non-crystallographic symmetry in protein structure refinement. Acta Crystallogr. D, Biol. *Crystallogr*. **52**, 842–857 (1996). 10.1107/S0907444995016477 15299650

