# Supplementary material for: The human IL-17A/F heterodimer: a two-faced cytokine with unique receptor recognition properties
Source: Sci Rep. 2017 Aug 21;7:8906. doi: 10.1038/s41598-017-08360-9 (PMC5566378; doi:10.1038/s41598-017-08360-9)
Supplement: Supplementary file 1 — Supplementary information [file 41598_2017_8360_MOESM1_ESM.pdf]

The human IL-17A/F heterodimer: a two-faced cytokine with unique receptor recognition properties

Arnaud Goepfert, Sylvie Lehmann, Emmanuelle Wirth & Jean-Michel Rondeau

Novartis Institutes for BioMedical Research, Novartis Pharma AG, CH-4002 Basel, Switzerland

Supplementary information

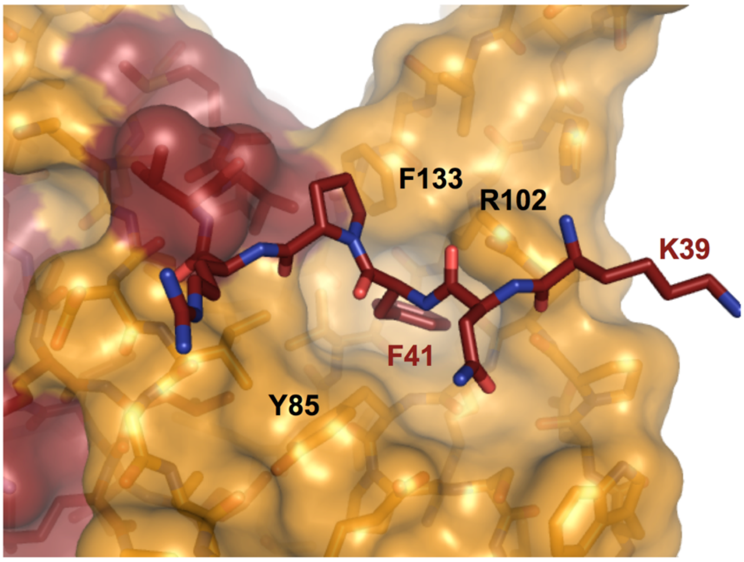

Supplementary Figure 1 Blockade of Site 1F of free IL-17A/F by Phe41 from the A-subunit. Close-up view showing a surface representation of the free IL-17A/F heterodimer. The F- and A-subunits are in orange and carmine, respectively. The N-terminal residues Lys39 to Arg43 of the A-subunit are shown in stick representation and not included in the surface representation of the cytokine for clarity. Note the binding of the side-chain of Phe41 into a pocket lined by Tyr85, Phe133 and Arg102 from the F-subunit. This pocket corresponds to the binding site for Trp62 of IL-17RA.

IL-17	Chain	Site 1	Site 2	Site 3
A	A	L49, I51, E80, E83, Y85, L122, **R124**, F133	**N55**, **S63**, **D65**, Y67, **R78**, V88, I89, P86, W90	K61, S64, Y66, **R69**
	A'	R43, L135	Q116, **Q117**, **E118**, K137, L139	L97, Y108, **H109**, **N111**, V113, V147, P149, I150, V151, H153, V154
F	F	I59, I61, W88, P90, N91, **Y93**, S95, V130, R132, **H134**, V139, F141	Q66, **R67**, S71, **N73**, **E75**, Y84, V86, P94, E96, V97, V98, V148	M70, **R77**, **R72**
	F'	S54, M55	Q124, **Q125**, **E126**, K145, L147	L105, I116, S117, **N119**, S120, V121, V155, P157, V158, I159, H160, H161, V162

IL-17	Chain	Site 1A	Site 1F	Site 2A	Site 2F	Site 3A	Site 3F
A/F	A	L49, I51, E80, E83, Y85, L122, R124, F133	V45	N55, S63, D65, Y67, R78, V88, I89, P86, W90	Q117, E118, K137, L139	K61, S64, Y66, R69	L97, Y108, H109, N111, V113, V147, P149, V151, H152, H153, V154
	F	G53	I59, I61,W88, P90, N91, **Y93**, S95, V130, R132, **H134**, V139, F141	Q94, Q95, E96, K115, L117	Q66, **R67**, S71, **N73**, E75, **Y84**, V86, P94, **E96**, V97, V98, V148	L105, I116, S117, N119, S120, V121, V155, P157, V158, I159, H160, H161, V162	M70, I74, **R72,** **R77**

Supplementary Table 1 Conserved binding sites in IL-17A, IL17F and IL-17A/F. Residues lining the IL-17RA binding sites 1-3 in IL-17A (4hsa.pdb), IL-17F (3jvf.pdb) and IL-17A/F (this work) are listed. While the two sides of the homodimeric cytokines are identical by symmetry, the "A-face" differs from the "F-face" in the heterodimeric IL-17A/F, hence Sites 1-3 are sub-divided into Sites 1A-3A and Sites 1F-3F. Residues lining Sites 1F-3F were directly identified from the crystal structure of the IL-17A/F complex with IL-17RA. Residues lining the "A-face" were listed based on the assumption that the binding of IL-17RA to the "A-face" of IL-17A/F is similar to its binding to IL-17A. Residues in bold case are engaged in H-bonded interactions with IL-17RA, while underlined residues form salt-bridges with the receptor.

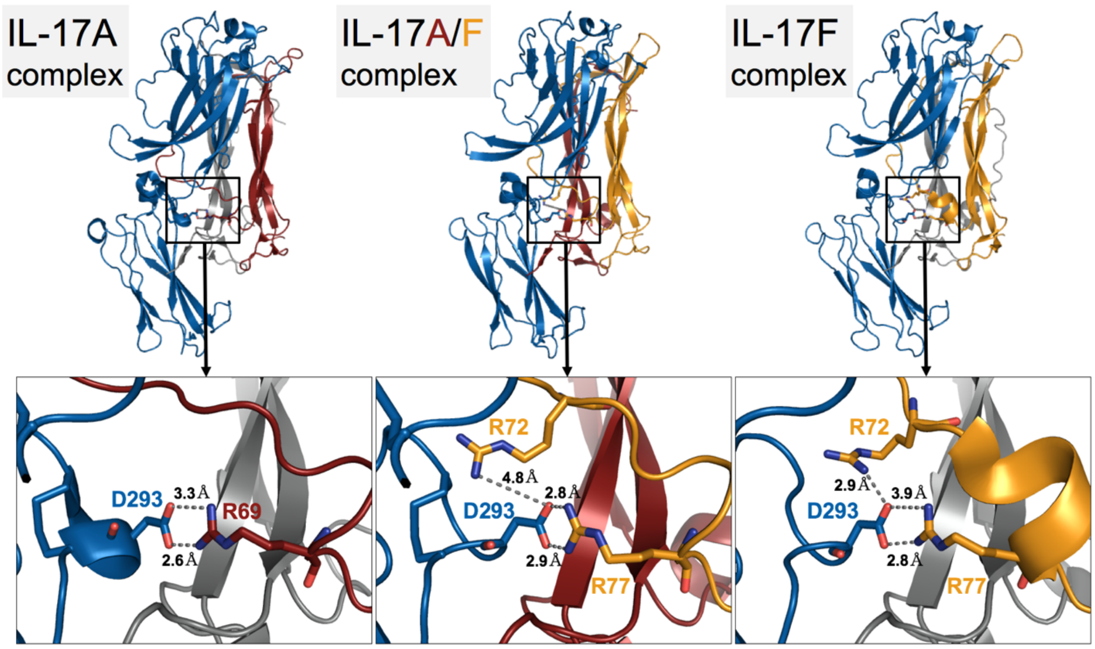

Supplementary Figure 2 Key salt-bridge interaction between IL-17RA (blue ribbon) and IL-17A (left view; the two IL-17A subunits are shown as a carmine and gray ribbon, respectively; figure based on 4hsa.pdb), IL-17A/F (center; carmine ribbon: A- subunit; orange ribbon: F-subunit; this work), and IL-17F (right view; the two IL-17F subunits are shown as an orange and gray ribbon, respectively; based on 3jvf.pdb). The top panel shows an overall view of the complex, the lower panel a close-up view of the salt-bridge interaction with the side-chains involved depicted in stick representation.

Cytokine	Variant	IL-17RA K_d (nM)	IL-17RC K_d (nM)
**IL-17A**	wt	0.7	0.4
	R69A	297	1500
**IL-17F**	wt	136	4
	R77A	726	905
**IL-17A/F**	wt	17.5	0.6
	A(R69A)	42	15
	F(R77A)	30	5
	A(R69A), F(R77A)	202	1100

Supplementary Table 2 Summary of equilibrium dissociation constants obtained from SPR analysis of wild-type and variant IL-17A, IL-17F and IL-17A/F binding to the IL-17 receptors IL-17RA or IL-17RC (see also Fig. 5). The K_d values shown are the mean from at least five independent experiments.

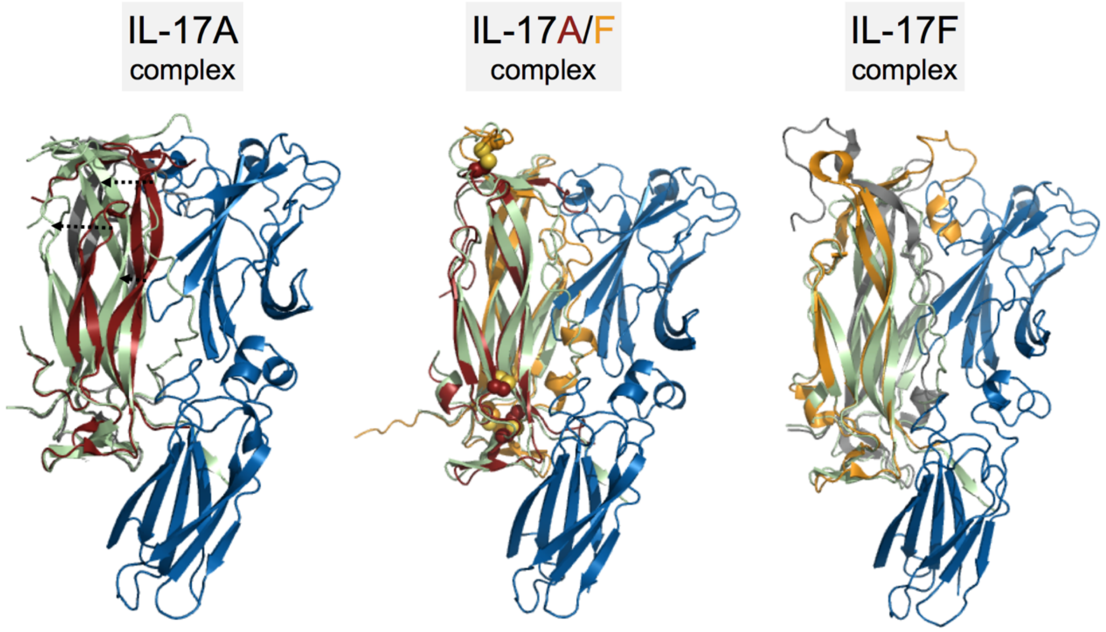

Supplementary Figure 3 IL-17RA (blue ribbon) induced allosteric changes in IL-17A (left view, figure based on 4hr9.pdb and 4hsa.pdb), IL-17A/F (center, this work) and IL-17F (right view; based on 1jpy.pdb and 3jvf.pdb). The receptor-bound cytokines are depicted as a green ribbon, while the free cytokines are shown in carmine/grey (IL-17A), carmine/orange (IL-17A/F A- and F-chain, respectively) and orange/grey (IL-17F). Structure-based alignments were computed with Lsqman[46] using a maximum matching distances of 1.2Å. Note the large allosteric changes observed with IL-17A, indicated by dotted arrows.

Maximum matching distance		IL-17A	IL-17A/F	IL-17F
**3.50Å**	rms deviation (Å)	1.52	1.08	1.14
	matched residues	132	192	192
**1.2Å**	rms deviation (Å)	0.56	0.72	0.69
	matched residues	81	141	133

Supplementary Table 3 Least square superpositions of the free- and IL-17RA-bound forms of IL-17A, IL-17A/F and IL-17F, calculated with Lsqman[46].

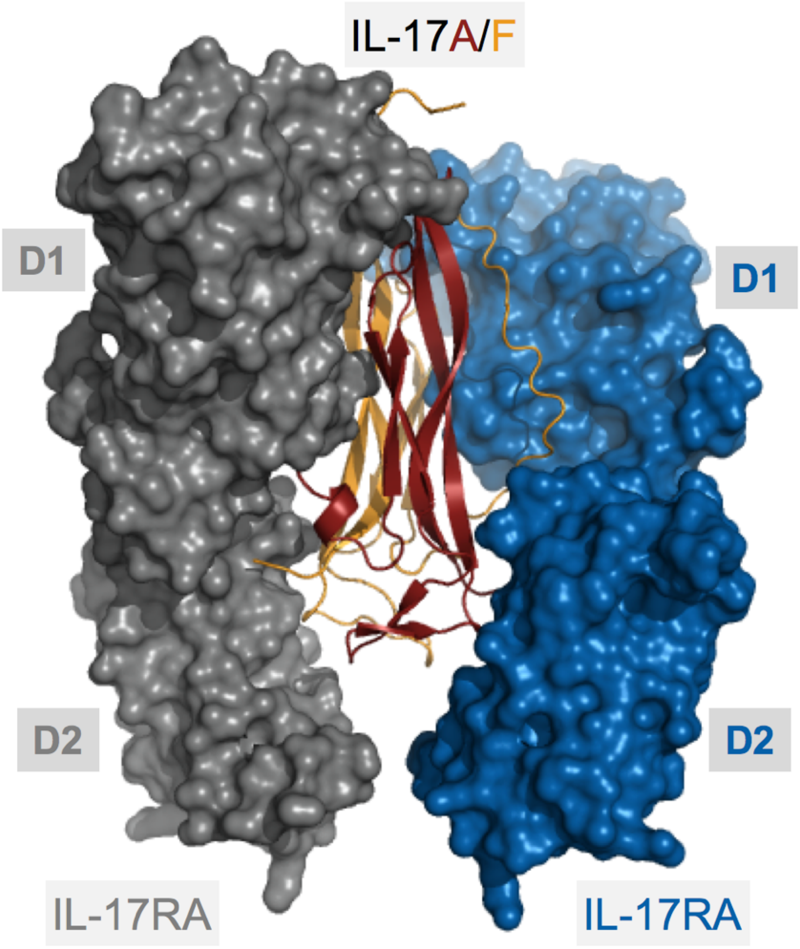

Supplementary Figure 4 Structural model of IL-17A/F (A-chain:carmine/F-chain:orange ribbon) bound to two copies of the IL-17RA ECD (blue and grey surface representations). The blue IL-17RA is based on the crystal structure of the IL-17A/F complex with IL-17RA bound to the "F-face" of the cytokine. The grey IL-17RA model was placed by applying the transformation that brings the F-chain onto the A-chain of the heterodimer to the blue IL-17RA model. Note that there is no steric clash between the two receptor chains.

**Model**	**Resolution limit (Å)**	**R_{work}**	**R_{free}**	**R_{free}-R_{work}**
**Model refined against 3.30Å data**	3.30	0.1856	0.2431	0.0575
	3.57	0.1851	0.2426	0.0575
	3.62	0.1839	0.2411	0.0572
**Model refined against 3.57Å data**	3.57	0.1951	0.2547	0.0596
**Model refined against 3.62Å data**	3.62	0.1922	0.2532	0.0610

Supplementary Table 4 Paired refinement tests showing that the model refined against 3.30Å data fits the diffraction data to 3.57Å or 3.62Å resolution better (lower R_{free}) than the corresponding models refined against 3.57Å or 3.62Å resolution data.
